# The effect of swearing on error-related negativity as an indicator for state disinhibition

**DOI:** 10.1177/17470218241308560

**Published:** 2025-01-13

**Authors:** Venja Beck, Joseph L Brooks, Richard Stephens

**Affiliations:** 1 Institute for Interdisciplinary Studies (IIS), University of Amsterdam, Amsterdam, The Netherlands; 2 School of Psychology, Keele University, Staffs, UK

**Keywords:** Swearing, state disinhibition, error related negativity, physical performance

## Abstract

Swearing has been linked to increased strength performance, and state disinhibition may be the mechanism linking swearing and strength. Error-related negativity (ERN) is a neural signal associated with response monitoring. Its reduction has been proposed as neural marker for state disinhibition, and therefore, we predicted that swearing would lead to a decreased ERN compared with neutral word repetition, indicating state disinhibition. The study (*N* = 52) used a within-subjects experimental design with two conditions. Participants repeated either a swear or neutral word aloud for 10 s before engaging in an arrowhead flanker task, a grip strength task, and several questionnaires. ERN was measured continually using electroencephalography (EEG). The study replicated previously found effects of swearing on strength, humour, positive emotion, and distraction. In addition, swearing was found to have a significant effect on state behavioural activation (BAS drive). However, results indicated no significant difference between conditions on ERN amplitude. This pre-registered study has confirmed that, relative to a neutral word, repeating a swear word leads to increased performance on a grip strength task while also confirming effects of swearing on positive emotion, humour, and distraction. Its novel contribution is confirming that swearing raises state behavioural activation. This supports application of Hirsh et al.’s state disinhibition theory to swearing to some extent, although the absence of any effect of swearing on ERN limits this interpretation.

## Introduction

### Swearing

Swearing is defined here as the use of taboo words, often associated with the expression of strong emotion (Vingerhoets et al., 2013). This definition is extended to include swear word repetition, as this is how it is often operationalised in studies of swearing, including the present study (e.g., Stephens et al., 2023). Swearing holds a unique position in language (Stapleton et al., 2022) with effects including increased pain tolerance and reduced subjective experience of pain (Robertson et al., 2017; Stapleton et al., 2022; Stephens et al., 2009). Swearing has also been associated with strength and power performance (Stephens et al., 2018, 2023). Swearing appears to be linked with increased autonomic nervous system (ANS) activity as measured through skin conductance rate (Harris et al., 2003; Huang & Nicoladis, 2020; Tomash & Reed, 2013) and heart rate (Stephens et al., 2009), thought to be indicatory of increased emotional arousal.

The effect of swearing on strength performance is particularly of interest here. Stephens et al. (2018) found repeating a swear word to have a beneficial effect on strength performance. In the second experiment of this study, 52 participants participated in a task which involved repeating either a chosen swearword or neutral word out loud, after which force on a handgrip task was measured. Force was significantly higher following swearing than following neutral word repetition (*p* < .001). These effects were replicated in (Stephens et al., 2023). However, these results were not linked to the hypothesised underlying mechanism linking swearing to physical performance, changes in ANS activation (Stephens et al., 2018). An alternate explanation for this effect might be found in state disinhibition.

### State disinhibition

Trait disinhibition has been defined as a broad personality trait reflecting individual differences in self-regulation or control of one’s behaviour, tending towards under-controlled rather than over-controlled (based on definition by Clark & Watson, 2008, as cited in Mullins-Sweatt et al, 2019). By extension, state disinhibition can be defined as temporarily tending towards behaviours that are under-controlled rather than over-controlled. Hirsh et al. (2011) used this construct in their theoretical framework as a mechanism to explain a range of social behaviours under conditions where state disinhibition may occur.

Hirsh’s theory of state disinhibition builds on the behavioural inhibition system (BIS), a neural/psychological system involved in the interruption or prevention of behaviours that may lead to negative outcomes, occurring in response to threats as well as response conflict (Gray, 1982). Its counterpart, the behavioural approach system (BAS), is responsible for the pursuit of potential positive outcomes (Gray, 1990).

The conceptualisation of BAS as a single construct as opposed to multiple related factors should briefly be addressed here. A commonly used scale for measuring trait BIS and BAS, the Carver and White (1994) scale, consists of three subscales measuring BAS; the drive, fun-seeking, and reward responsiveness subscales. Drive measures the drive one experiences to pursue goals persistently; fun-seeking measures willingness to spontaneously follow potential rewards and desire for new rewards; while reward responsiveness measures positive responses to potential rewards. These scales are considered to measure three separate factors which map onto BAS reward. There is some debate surrounding the separation of BAS into these factors (Maack & Ebesutani, 2018), and different studies appear to favour different subscales as being (most) representative of BAS sensitivity (Carver & White, 1994; Keough et al., 2017; Taubitz et al., 2015). For this study, we will consider BAS to be a multidimensional construct, consistent with the original conceptualisation of the BAS (Carver & White, 1994; Gray, 1990).

State disinhibition then, refers to a temporary reduction in activity of the BIS, leading to reduced response conflict and an inclination towards performing the most salient actions. Hirsh et al. (2011) describe this as occurring through three distinct theoretical pathways.

The first route to state disinhibition happens through BAS activation. As it is associated with the pursuit of a particular goal, resources are oriented towards the most salient goal, and less attention is paid to competing potential responses. It is proposed that this results in silencing of the BIS and thus, state disinhibition (Hirsh et al., 2011).

The second route occurs through depletion of cognitive resources. The more cognitive resources are available, the more attention can be given to various potential responses. Conversely in situations where one’s cognitive resources are more limited, this becomes less feasible, and limited attention is instead deployed to the most salient possibilities. It is proposed that this leads to state disinhibition, as there are simply not the resources to face conflicting responses, and conflict-related BIS activity is decreased. Examples of situations decreasing the cognitive load include distraction, heightened cognitive load, and reduced cognitive processing induced by alcohol intoxication (Hirsh et al., 2011).

In the third pathway, disinhibition is induced through the reduction of social desirability concerns. People will often adapt their behaviour to be in line with social acceptability, despite it potentially being in conflict with internal goals. In situations where social desirability becomes less relevant, such as anonymity, it is proposed that the evaluation of the social acceptability of a set of actions might be reduced, leading to selection of a salient response option, regardless of how this action might be perceived (Hirsh et al., 2011).

### Disinhibition and swearing

Hirsh et al.’s (2011) theory of state disinhibition provides a theoretical framework to explain the effects of swearing in the context of strength performance (Stephens et al., 2018, 2023). State disinhibition, as described above, is characterised by reduced response conflict and attention directed towards the most salient response options. If swearing indeed triggers a state of disinhibition, it would be followed by a redirection of resources towards the most salient behaviour. During a grip strength test, this could increase attention towards the grip strength, while reducing attention towards monitoring processes that may prevent excessive force (Hirsh et al., 2011; Stephens et al., 2023). This line of reasoning could explain the increased strength performance found following swearing (Stephens et al., 2018, 2023).

Stephens et al. (2023) detail how each of the three pathways described in Hirsh et al. (2011) might link swearing to state disinhibition. The pathway through BAS activation is described as the “hot cognitions” pathway. Swearing may act as a “hot cognition,” which has been shown to lead to BAS activation, in turn silencing BIS activity. Regarding the depletion of cognitive resources, swearing could trigger this pathway by causing some level of distraction and increasing cognitive load, leaving reduced cognitive resources to allow for the monitoring of various actions. The social desirability pathway is also a good candidate for causing state disinhibition. Due to the taboo nature of swearing, the act already violates social desirability somewhat, which may reduce social desirability as a concern after the fact (Stephens et al., 2023).

Stephens et al. (2023) provides some support for a link between swearing and state disinhibition, as various factors associated with state disinhibition were found to be increased after repeating a swear word, such as risky behaviour, humour, distraction, and self-confidence. Whether these factors are part of a pathway connecting swearing and strength is less clear. Most of the factors did not show a (significant) mediating effect, with exception of humour, which was found to be an important mediator of the swearing-strength relationship. This is thought to be evidence particularly in favour of the “hot cognitions” pathway, as humour might activate the BAS triggering disinhibition.

State BIS/BAS may be measured using an adapted version of the Carver and White (1994) 20-item trait BIS/BAS scale. Van den Bos et al. (2009) deployed a state version with modification to the wording of each item so that it was oriented to the immediate moment rather than in general. Like the trait scale, the state scale measures BIS and three subcomponents of BAS: BAS reward responsiveness; BAS drive, and BAS fun-seeking.

### ERN and ERPs

An event-related potential (ERP) is the neural response to a specific event, like a stimulus, or in our case a participants’ response (Luck, 2014). This response is extracted from electroencephalography (EEG) data, in which residual electrical activity from the brain is measured at the scalp. By averaging the EEG data of multiple instances of the same event together, it is possible to extract the activity directly related to the event, as noise from other activity would cancel out. This constitutes the ERP (Luck, 2014).

**Figure 1.**
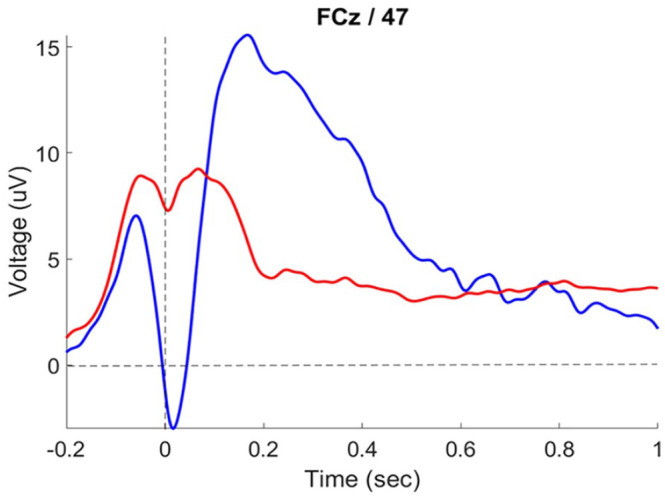
Response-locked ERP at FCz on arrowhead flanker task. Blue line shows incorrect trials, with the peak of the ERN occurring shortly after 0. Red line shows ERP of correct trials.

The ERN is a particular ERP component that occurs as a negative peak shortly after a mistake is made, generally within 100 ms of response (Gehring et al., 2018). Figure 1 shows what this component looks like. The ERN is usually recorded at the frontocentral regions of the scalp (de Bruijn et al., 2020; Klawohn et al., 2020). The ERN is thought to reflect activity of a response monitoring system which picks up on conflicts between the planned and actual responses, as well as conflicts between multiple competing responses. The commission of an error represents a mismatch between the intended and actual response which is detected by the response monitoring system, which can be measured as the ERN.

The anterior cingulate cortex (ACC) is thought to be the point of origin of the ERN. ACC activity is associated with conditions with higher response conflict, and its location near the corpus callosum corresponds to the frontocentral regions where the ERN is measured (Devinsky et al., 1995; Luck, 2014; Yeung et al., 2004). Hirsh et al. (2011) suggests that the ACC might act as a “cortical extension” of the behavioural inhibition system. Research by Migliorini et al. (2015) seems to support this notion, as they found the ACC area to be linked to inhibition performance. In addition, Amodio et al. (2008) found higher trait BIS scores to be significantly correlated with larger ERN amplitudes. Boksem et al. (2006) also found higher BIS scores to be associated with larger ERN amplitudes. Interestingly scores for BAS were not found to be linked to ERN amplitude in this study. As higher BIS scores are related to increased ERN amplitude, indicating response and conflict-monitoring, we would expect that in a state of disinhibition we would see the opposite effect, reduced conflict-monitoring and thus a reduced ERN amplitude.

Indeed, this reduction in ERN amplitude has been found in some studies which tested participants under the influence of alcohol, an expected disinhibitor. Ridderinkhof et al. (2002) conducted a double-blind study in which 14 male participants participated in three sessions where they were given either a low dose of alcohol, a high dose, or a placebo. They were then tested on a flanker task, in which they completed 16 blocks of 110 trials per session. ERN was measured as the peak negativity between 0 and 150 ms after the participant’s response, this signal was strongest frontocentral electrodes. Analysis shows that alcohol consumption significantly affected ERN amplitude, which was reduced in both low- and high-dose conditions. This result, according to the author, may be explained by a reduction in action monitoring, or lapses in the ACC function.

A further study on acute alcohol consumption and ERN conducted by Easdon et al. (2005) provided similar results. They studied three groups of eight participants assigned to low dosage of alcohol, moderate dosage, or placebo. Participants were tested on a go/no-go task both before and after alcohol/placebo consumption. Three or four blocks of 300 trials were done before consumption, and four blocks after consumption. ERN peaked around 50 ms postresponse, and the signal was largest at midline central scalp regions. ERN was reduced significantly post-consumption in both alcohol groups, while in the placebo group it was comparable pre- and postconsumption. Easdon et al. (2005) also recorded the error positivity (Pe), a smaller peak that appears later in response to erring, which peaked between 200 and 300 ms. Pe was also reduced following alcohol consumption.

In conclusion, the ERN may occur as a result of conflict-monitoring processes. The expectation is that the amplitude of the ERN would be reduced in state disinhibition, reflecting a reduction in activity of response monitoring processes.

### The present study

Here, we aimed to both replicate the effect of swearing in hand grip strength found in Stephens et al. (2023), and augment it with additional measures for state disinhibition; the ERN and a state BIS/BAS scale. The hypotheses and methodology were preregistered (see https://aspredicted.org/JL3_PS1).

The study comprised an experiment comparing effects of repeating a swear word with repeating a neutral word (as a control condition). Strength was measured using in an isometric hand-grip strength task after participants repeated a swear word. The grip strength task was followed by a set of additional measures associated with state disinhibition: Visual Analogue Scales for positive emotion, humour, negative emotion, distraction, and novelty; flow state measured using the Engeser (2012) and Ulrich (Ulrich et al., 2014) flow scales; state confidence and anxiety measured using the Revised Competitive State Anxiety II (Cox et al., 2003). These measures were used in Stephens et al. (2023) as potential mediators in the “hot cognitions” pathway of disinhibition.

On top of these, two novel measures were deployed. These were an adapted version of the trait BIS/BAS scale developed by Carver and White (1994) was used to measure state BIS/BAS, scores of which could similarly be mediators of the strength-swearing relationship, serving as indicators of state disinhibition.

The other novel measure was the ERN, which was induced using the arrow version of the Eriksen flanker task (Eriksen & Eriksen, 1974; Stoffels & Van der Molen, 1988).

### Hypotheses

Based on the described aims of the study our hypotheses are as follows: When comparing swear word repetition to neutral word repetition we expected to see (1) increased handgrip task performance, (2) increased errors on incongruent flanker items, (3) decreased ERN amplitude, (4) increased state BAS score, (5) decreased state BIS score, (6) increased flow, (7) increased positive emotion and humour, (8) decreased negative emotion, (9) increased distraction and novelty, (10) increased state self-confidence, (11) decreased cognitive anxiety, and (12) increased somatic anxiety. It was further hypothesised (13) that the predicted beneficial effect of swearing on physical task performance would be mediated by ERN scores.

## Methods

### Participants

The majority of participants comprised psychology undergraduate students who received partial course credit for participating in this study. Other participants were recruited through word of mouth.

Using G*Power 3.1.9.7 (Faul et al., 2007), a power analysis was conducted to estimate sample size. We used an effect size of 0.428, based on de Bruijn et al. (2020) (*N* = 27). Using α = .05 and power = .80, the minimum sample size was found to be 45.

In total 55 participants were recruited for the study, of which two were pilots who did not complete the full final version of the experiment, and were thus not included. One further participant was excluded due to premature termination of the study. In the sample of *N* = 52 participants included in the study, the mean age was 21.135 (*SD* = 6.020). The sample consisted of 13 male, 37 female, and 2 non-binary participants. Six participants reported being left-handed, the other 46 reported being right-handed. For each participant it was determined randomly whether they would start with the swear or the neutral condition.

Of the included participants, 30 started with the neutral condition and 22 with the swearing condition. Seven participants were excluded from the ERN analysis due to bad or incomplete EEG data (*N* = 45). Due to errors in answer notation, one participant was excluded from the grip strength task and two participants were excluded from all questionnaires. One additional participant was removed from the confidence scale, and one from the BAS drive scale. Twenty-two further participants were excluded from the BAS fun-seeking scale, of which the responses were not recorded due to a technical error.

Informed consent was obtained from each participant prior to starting the study. Ethical approval for the study was granted by the Keele University Psychology Ethics Committee.

### Materials

#### Vocalisations

For the vocalisations or word repetition phase of the experiment, participants were asked to repeat a word out loud for 10 s and were provided with the countdown. The words used were chosen by the participant and were different for the two conditions. In the swear condition, participants were asked to use a word chosen by the prompt “a swearword you might use if you hit you head or stubbed your toe.” In the neutral condition, the word was one chosen by the participant based on the prompt “a word you might use to describe a table.” The two most common swearwords used were “shit” or “fuck.” The two most common neutral words were “flat” and “wood(en).”

#### EEG setup

EEG recordings were made using a 24-bit DC-coupled Biosemi ActiveTwo System (Biosemi; Amsterdam, Netherlands), running at a 1024 Hz sampling rate. Data were recorded across 64 active silver/silver chloride scalp electrodes, attached to a cloth cap in 10-10 system electrode position (Seeck et al., 2017). Three EOG electrodes were placed on the skin (below right eye, next to right, and left eyes). An additional two electrodes were placed on the mastoids, one on either side. SignaGel (Parker Labs; https://www.parkerlabs.com/) was used as the electrolyte gel. All electrodes were adjusted to have offsets within a range of -10 to 10 mV of the common mode voltage. The Biosemi system does not require electrode impedance checks. Data were recorded relative to the CMS/DRL circuit and re-referenced offline (see data analysis methods below). Initial low pass filtering was performed in the Analogue-to-Digital Converter’s decimation filter which has a fifth-order sinc response with a –3 dB point at 1/5th of the sample rate.

#### Hand dynamometer and grip strength task

The JAMAR^®^ hand dynamometer (Lafayette Instruments, Lafayette, IN) was used to measure isometric hand grip strength in kilogrammes in the participant’s dominant hand. In the grip strength task participants were instructed to pick up the dynamometer in their dominant hand and squeeze it for 3 s as hard as they could. After the 3 s, they read off and wrote down the force in kilogrammes.

#### Visual analogue scales

Participants were asked to rate perceptions on a series of visual analogue scales (VAS), on a scale from 1 to 100, where 1 indicated *they did not experience the stated perception* and 100 indicated *they strongly experienced the perception in question*. They were asked to rate experiences of positive emotion (“Repeating the word made me feel a positive emotion along the lines of excitement or happiness”), negative emotion (“Repeating the word made me feel a negative emotion along the lines of anger or sadness”), humour (“Repeating the word was funny or humorous”), distraction (“Repeating the word distracted me from thinking about other things”), and novelty (“Repeating the word felt like a new or different experience”).

#### State BIS/BAS

A modified version of the Carver and White (1994) (trait) BIS/BAS scale was used to measure state BIS and BAS. The BAS scale consists of three BAS subscales: reward responsiveness, drive, and fun-seeking. The BIS scale consisted of seven statements, the BAS reward responsiveness of five statements, BAS drive of four statements, and BAS fun-seeking of four statements, for a total of 20 items. All statements had to be rated by the participant on a 7-point Likert-type scale (1 = *strongly disagree*, 7 = *strongly agree*). A high score was indicative of a high BIS or BAS sensitivity respective to the appropriate. To adapt the scale to be suitable for state BIS/BAS measurements, statements were changed to refer to the current moment, as was done in Van den Bos et al. (2009). For example, “I have very few fears compared to my friends.” (Carver & White, 1994, p. 323) becomes “At this moment, I feel like I have very few fears compared to my friends.” Van den Bos et al. (2009) reported reliability of α = .76 on their adapted state BIS scale and α = .76 on the state BAS scale.

#### Revised Competitive State Anxiety II

The Competitive State Anxiety II (CSAI-2R) consists of 17 statements describing feelings of confidence and anxiety, divided up into three subscales: somatic anxiety, cognitive anxiety, and self-confidence (Cox et al., 2003). Participants were asked how well each statement matched how they felt during the grip task on a 4-point Likert-type scale (1 = *not at all*, 4 = *very much so*). All subscales were found to be reliable at α > .80 (Cox et al., 2003).

#### Flow scales

Two flow scales were used in this study. The Engeser Flow Short Scale (Engeser, 2012; Engeser & Baumann, 2016) consists of 10 items rated on a 7-point Likert-type scale. The Likert-type scale ranges from “not at all,” scoring 1 point, to “very much,” which scores 7. A high score indicates high flow. The scale has good reliability (α = .92) (Engeser & Baumann, 2016). Three items from the Ulrich Flow Scale were used to measure enjoyment on a 7-point Likert-type scale, where 1 corresponds to “I do not agree at all” and 7 to “I completely agree” (Ulrich et al., 2014). Ulrich et al. (2014) reported an acceptable reliability of α = .80. Instructions for both flow scales requested the participant to rate these items based on how they experienced the hand grip task.

#### Flanker task

The arrowhead version of the Eriksen flanker task was used to elicit the ERN (Eriksen & Eriksen, 1974; Stoffels & Van der Molen, 1988). In this task, participants were shown a set of five arrows in a row horizontally. Participants were instructed to respond to these by indicating which direction the middle arrow was pointing using the “f” (left) and “j” (right) keys on a keyboard. The middle arrow pointed either left or right, and the flanking arrows pointed either in the same (congruent) or opposite (incongruent) direction of the middle arrow. Each type of trial; congruent left (<<<<<), congruent right (>>>>>), incongruent left (>><>>), and incongruent right (<<><<), was shown the same number of times within a block. For the timing we followed the methodology of de Bruijn et al. (2020). Within each trial, participants were first shown a fixation cross for 1000 ms, then a 250-ms blank screen. Followed by the presentation of arrows for 100 ms, and a 900-ms maximum response window, which terminated early with the participant’s response. For the inter-trial interval (ITI) we deviated from the methodology of (de Bruijn et al., 2020). Instead of a fixed window, the duration of the ITI was randomised for each trial and ranging between 300 and 600 ms.

### Procedure

After consent was obtained, electrodes were placed on participants’ mastoids, after which they put on the cloth cap, into which gel and 64 scalp electrodes were placed. Alongside the EEG set up procedure, participants were given instructions for the tasks and asked to come up with their swear word and neutral word.

Once the EEG set up was completed participants completed 10 practice trials for the flanker task, in which they received feedback after every trial. If so desired, participants were able to do another 10 practice trials. When the practice trials were completed, the experimenter left the room, and the experiment proper was started. The experiment was built in PsychoPy-2022.2.4 (Peirce, 2007).

**Figure 2.**
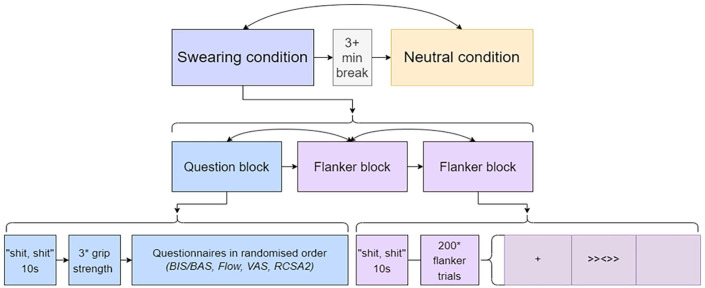
Overview of study design.

Figure 2 shows an overview of the study design. The experiment consisted of two conditions, which both contained three blocks: two flankers, one questionnaire. All blocks belonging to the same condition were grouped together. The order in which conditions appeared (swear first or neutral first) was randomised, as was the order of blocks within each half of the study. Participants could take a break between each block for however long they wished, and between condition they took a minimum 3-min break, after which they could continue when they wanted.

Flanker blocks started with the instructions for the flanker task:

You will be shown a set of 5 arrows. Please indicate which direction the **middle** arrow is pointing as quickly and accurately as you can. If the arrow is pointing left (<) press “f.” If the arrow is pointing right (>) press “j.”

This was followed by the vocalisations, with the instructions for swear or neutral word repetition depending on the condition. After the instructions, participants were continuously shown 200 flanker trials, 50 of each type in a random order, with a 5-s break halfway for participants to rest their eyes. At the halfway point before the break and at the end of the block, participants were provided with feedback aimed to encourage a desirable proportion of errors. If the accuracy on the preceding trials was below or equal to 75%, participants were asked to “Please try to be more accurate.” If it was above 90%, they were asked to “Please try to respond faster.” Otherwise, they were told “You’re doing a great job.”

Questionnaire blocks started with a brief introduction informing the participant that the upcoming block would consist of questionnaires, with the additional warning that participants should ensure that they filled out every questionnaire completely. After this came the vocalisations. Word repetition was always immediately followed by three instances of the grip strength task. Following the grip strength task, the Engeser flow short scale, Ulrich flow scale, state BIS/BAS questionnaire, VAS, and CSAI-2R were presented in a randomised order.

At the end of the experiment, participants were asked what they thought the experiment was testing for, and in later instances were also asked if they had to inhibit swearing during the flanker task.

### Analysis

#### Data processing

As preregistered, outliers were defined as scores more than three times the interquartile range above the 75th percentile value defined at the upper end, and scores more than three times the interquartile range below the 25th percentile value at the lower end. Before the analyses, outliers were identified using JASP boxplots and then winsorised. Winsorisation percentiles are included in the descriptives in Table 1. To preserve as much data as possible, items on questionnaires were averaged together, excluding missing values, rather than added up. Thus, means represent the average item score on the relevant questionnaire. The following items were reverse scored: Items 5 and 7 on the state BIS subscale, and Item 14 of the CSAI-2R, which falls under the somatic anxiety scale. These item scores were reversed before averaging.

**Table 1.** Descriptive values.

	Swearing				Neutral			
	Mean	*SD*	Valid	Percentile	Mean	*SD*	Valid	Percentile
**Strength (kg)** *	29.014	8.035	51	98th	27.617	5.984	51	90th
Number of Errors (flanker task)	32.327	15.522	52	-	32.423	17.179	52	94th
Reaction time (s) (flanker task)	0.300	0.042	52	-	0.308	0.048	52	96th
ERN peak-to-peak	13.017	6.375	45	-	12.714	6.433	45	96th
State BAS reward	4.535	0.823	50	-	4.457	0.912	50	-
**State BAS drive** **	3.294	1.094	49	98th	1.868	0.766	50	-
State BAS fun	3.863	1.242	28	-	3.810	0.920	29	97th
State BIS	3.889	1.321	50	-	4.107	1.104	50	-
Engeser Flow	3.552	0.796	50	-	3.493	0.784	50	98th
Ulrich Flow	3.473	1.006	50	-	3.253	1.134	50	-
**Positive VAS** **	3.739	2.539	50	-	2.765	2.278	50	-
**Negative VAS** *	1.959	2.140	50	98th	1.304	1.448	50	-
**Humour VAS** ***	6.379	2.884	50	-	3.791	2.673	50	-
**Distraction VAS** *	7.052	1.993	50	-	6.163	2.621	50	-
Novelty VAS	4.565	2.811	50	-	4.977	2.741	50	-
Somatic Anxiety	0.490	0.387	50	94th	0.551	0.360	50	96th
Cognitive Anxiety	0.888	0.566	50	-	0.966	0.738	50	-
Confidence	1.487	0.646	50	-	1.433	0.594	49	-

For difference in means between swearing and neutral: *p < .05 **p < .01 ***p < .001.

#### EEG data processing and ERN quantification

EEG data were processed in MATLAB using the FieldTrip (version 20230118) toolbox (Oostenveld et al., 2011).

All recorded EEG data sets were reference to the average mastoids and bandpass filtered using a 0.01- to 30-Hz band pass filter, after which the data were epoched into –500 to 1000 ms segments around the response (de Bruijn et al., 2020; Klawohn et al., 2020). Noisy channels were interpolated. However, the current study only looks at FCz, which is commonly used for measuring the ERN (de Bruijn et al., 2020; Klawohn et al., 2020; Nigbur & Ullsperger, 2020). This electrode was not subject to interpolation in any participant. Nonblink artefacts were taken out manually using the summary view in FieldTrip, where trials with a comparatively high variance were removed. Eye blinks and movements were removed using Independent Component Analysis (ICA) and manual selection and removal of blink components. They were identified by comparing artefacts to recordings from the EOG electrodes. On average 1.2 ICA components were removed per participant.

The minimum number of errors required in each condition was set at 6 (Olvet & Hajcak, 2009). One participant was taken out of the data set at this stage as there were not enough error trials in the data set after data cleaning. Four more were excluded due to substantially incomplete or otherwise unusable data. On average, there were 30.3 epochs contributing to the swearing error condition (min = 9; max = 64; *SD* = 15.1) and 30.7 epochs in the neutral error condition (min = 9; max = 83; *SD* = 17.3).

To construct ERPs, all segments corresponding to correct responses and incorrect responses were averaged for each participant. ERNs were quantified using peak-to-peak measurement on incorrect (error) trials only (congruent and incongruent), subtracting the negative polarity peak of the ERN deflection from a smaller preceding positive peak (de Bruijn et al., 2020; Klawohn et al., 2020; Sandre et al., 2020). The positive peak was identified by finding the highest peak between –120 to –5 ms, the negative peak by finding the lowest peak between –5 and 120 ms. These windows fall earlier than pre-registered, in which we expected to find the positive peak between –80 ms and 80 ms, and the negative peak between 0 and 150 ms. This deviation from the pre-registration was based on a pre-analysis visual inspection of the individual participant ERP waveforms which indicated that several participants had positive and negative peaks which fell outside of the pre-registered window. We can’t be sure why this was the case. However, exact timing of components can vary across studies due to various factors (e.g., Kappenman & Luck, 2012). Nonetheless, it was clear that the morphology of the waveforms was as expected and consistent with previous ERN studies. Thus, to resolve this issue, we estimated temporal region-of-interest windows for our data by identifying the positive and negative peaks on the aggregate grand average (i.e., grand average collapsed across conditions). We then placed 125 ms windows around these peaks which maximised the number of participants with peaks falling within the windows. This approach for locating regions of interest based on the “aggregate grand average” has been shown not to inflate Type 1 error rate despite being based on the collected data (Brooks et al., 2017). This process led to the windows that we used. At this stage, two more participants were excluded from the data set due to unclear presence of peaks based on the agreement of three experimenters. No baseline correction was applied because the peak-to-peak ERN measurement used here is a baseline-independent measure (Klawohn et al., 2020).

#### Analyses

All analyses were conducted in JASP 0.17.1, with exception of the mediation analysis which was conducted in R 4.2.2.

Confirmatory, preregistered analyses entailed conducting one-way repeated-measures analysis of variance (ANOVA) for Hypotheses 1 through 12, and mediation analysis of swearing on strength through ERN. The latter was conducted in R using the script written by Stephens et al. (2023), based on the MEMORE methodology of (Montoya & Hayes, 2017).

These analyses were augmented with a several exploratory analyses that were not preregistered. All ANOVA analyses mentioned above were conducted a second time, but with the condition order added as a between subject factor. This was to check for the presence of order effects as indicated by interactions. Any interactions found were further explored with a set of contrasts. Significant findings of this analysis can be found in the online supplementary materials. In addition, an exploratory mediation analysis of swearing on strength through BAS Drive was conducted.

## Results

Descriptive values can be found in Table 1. Hypothesis 1 was supported in confirmatory analysis as shown by the higher mean grip strength in the swear condition compared with the neutral condition, *F*(1, 50) = 4.66, *p* = .036, η_p_^2^ = 0.085. Hypothesis 2 predicted that more errors would be made in the swearing condition compared with the neutral condition. This was not supported in confirmatory analysis, as the difference in mean errors per condition was negligible, *F*(1, 51) = 0.003, *p* = .954, η_p_^2^ < 0.001.

**Figure 3.**
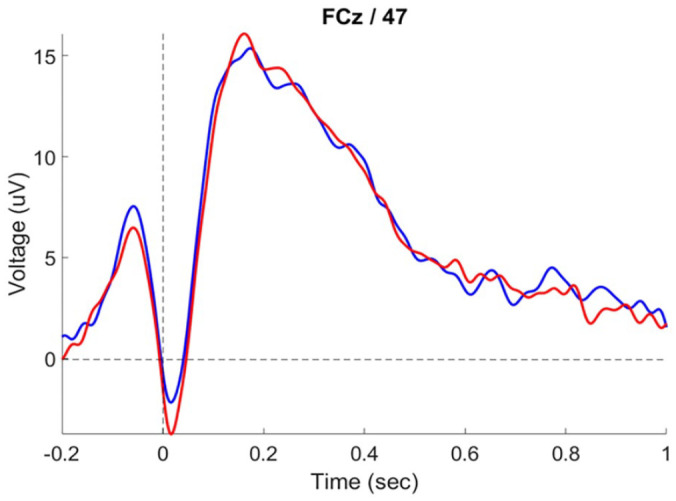
Grand average waveforms of incorrect trials, showing the ERN in the swearing (blue) and neutral (red) condition at FCz.

Hypothesis 3 predicted a reduced peak-to-peak value of the ERN in the swearing condition, compared with neutral. The waveforms of the ERN for each condition are shown in Figure 3. The hypothesis was not supported by confirmatory analysis, *F*(1, 44) = 0.115, *p* = .736, η_p_^2^ = 0.003.

Hypotheses 4 and 5 pertain to the state BIS and BAS scales. Hypothesis 4, which predicted an increase in state BAS in the swearing condition, was supported in confirmatory analysis. BAS Drive was significantly higher in the swearing condition, *F*(1, 48) = 185.385, *p* < .001 η_p_^2^ = 0.795. However, BAS reward responsiveness was not significantly increased in the swear, compared with the neutral condition, *F*(1, 49) = 0.789, *p* = .379, η_p_^2^ = 0.016. BAS fun-seeking also showed no significant difference across conditions, *F*(1, 27) = 0.157, *p* = .695 η_p_^2^ = 0.006. It should be noted that the fun-seeking subscale was not recorded in the first 23 participants due to a technical issue.

Hypothesis 5posited that state BIS would be decreased following swearing, compared with neutral word repetition. The hypothesis was not supported in confirmatory analysis as the mean difference between conditions was not significant, *F*(1, 49) = 3.082, *p* = .085, η_p_^2^ = 0.059.

We hypothesised that swearing would increase flow compared with neutral word repetition (Hypothesis 6). Flow was measured through two subscales, neither of which showed a difference between conditions in confirmatory analysis, thus not supporting the hypothesis. There was no effect for the Engeser short flow scale, *F*(1, 49) = 0.324, *p* = .572, η_p_^2^ = 0.007, nor for the Ulrich flow scale, *F*(1, 49) = 2.829, *p* = .099, η_p_^2^ = 0.055.

Hypothesis 7 was supported in confirmatory analysis as both the VAS for positive emotion, *F*(1, 49) = 10.121, *p* = .003, η_p_^2^ = 0.171, and the VAS for humour, *F*(1,49) = 65.432, *p* < .001, η_p_^2^ = 0.572, showed a substantially increased means in the swearing condition. Hypothesis 8, however, was contradicted by the data, as confirmatory analysis showed an increased negative emotion for the swear condition, *F*(1, 49) = 6.568, *p* = .014, η_p_^2^ = 0.118. Hypothesis 9 was partially supported in confirmatory analysis, as the mean score for distraction was higher in the swear condition, *F*(1, 49) = 5.782, *p* = .020, η_p_^2^ = 0.106. The results for novelty were not significant, *F*(1, 49) = 1.207, *p* = .277, η_p_^2^ = 0.024.

Hypothesis 10 was not supported in confirmatory analysis as no difference in confidence was recorded by the CSAI-2R, *F*(1, 48) = 0.865, *p* = .357, η_p_^2^ = 0.018. Confirmatory analyses also did not support any effect on cognitive anxiety (Hypothesis 11), *F*(1, 49) = 1.192, *p* = .280, η_p_^2^ = 0.024, or somatic anxiety (Hypothesis 12), *F*(1, 49) = 1.144, *p* = .290, η_p_^2^ = 0.023.

### Mediation analysis

**Figure 4.**
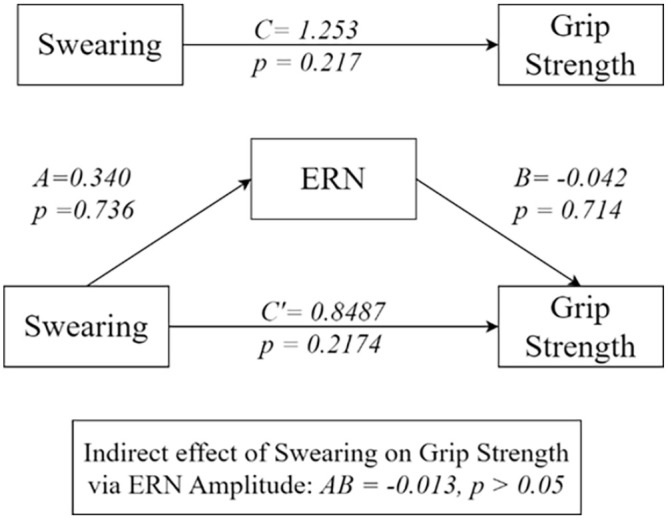
Mediation ERN.

Repeated measures mediation analysis was carried out using the MEMORE method developed by Montoya and Hayes (2017) implemented in R code. In the estimation of the 95% CI around the indirect effect, 5,000 bootstrapped samples were calculated. The results of the mediation analyses are shown in Figure 4. Hypothesis 13 was not supported in confirmatory analysis, as there was no indication of mediation effect from swearing on strength through ERN scores. As mentioned above, due to missing EEG data this mediation analysis includes a subset of *N* = 45 of the total *N* = 52 datapoints for grip strength. Because of this the direct effect of swearing on grip strength was not found to be significant here.

## Discussion

Here, we applied an experimental methodology using EEG to assess the theory that the effects of swearing on strength arise via state disinhibition. Several pre-registered hypotheses consistent with the state disinhibition theory were confirmed by the data.

Our expectations for this study were built on Hirsh et al.’s (2011) state disinhibition framework and previous findings by Stephens et al. (2018, 2023). In these studies, state disinhibition was used as the conceptual framework explaining the link between swearing and strength performance. In theory, we expect swearing to briefly bring about a disinhibited state, in which response conflict (state BIS) is reduced and consequently there is greater certainty of application towards the most salient action, which in the context of the experiment, is the hand grip task. This allows for a more targeted dedication of resources towards the salient action, resulting in increased strength performance. The reduction of response conflict via BIS de-activity can occur through various pathways, most notably through activation of the BAS system, as BAS-associated constructs were found to mediate swearing and strength performance in Stephens et al. (2023), referred to as the hot cognitions pathway. The ERN ties into this framework as a possible measure for state disinhibition, as the ERN is considered an indicator of response conflict. Here, we will consider our current findings and how they fit into this theoretical framework.

We confirmed our first hypothesis, finding a significant increase of roughly 1.4 kg in hand grip strength following swear word repetition compared with following neutral word repetition. This replicates previous work establishing the link between swearing and strength performance (Stephens et al., 2018, 2023), and lays the foundation for further explorations of this link.

Based on the state disinhibition framework, and in particular the hot cognitions pathway, we hypothesised (Hypothesis 4) that swearing would lead to relatively higher state BAS scores. This hypothesis was confirmed for the BAS drive subscale as we found a significant effect for swearing as well as a large effect size. We did not find effects for the BAS reward responsiveness subscale, or the BAS fun-seeking scale, although in the case of the latter, we had a reduced sample on this subscale due to a programming error, which would have impacted statistical power. However, the results from the BAS drive scale indicate increased BAS activity following swearing, consistent with the hot cognitions pathway for inducing state disinhibition via the Hirsh et al. (2011) state disinhibition framework.

Based on these results, one would expect that the increased state BAS would go hand in hand with reduced state BIS in the swearing condition (Hypothesis 5), as BIS and BAS are generally considered to be antagonistic systems {Corr, 2002 #145}{Hirsh et al., 2011 #45}. Confirmatory analysis found no main effect of swearing on state BIS score. This is unexpected, not only as the absence of evidence here is inconsistent with our hypothesis, but also because it contrasts with the observed increase in state BAS. An exploratory analysis (covered in Supplementary Materials) found an interaction with condition order such that state BIS was found to be significantly lower in the swearing condition compared with the neutral word condition when swearing occurred second; this was not found to be the case in the opposite direction. This could be interpreted as a reduced state BIS through swearing, present when swearing was the second condition, but not present when neutral was the second condition due to a carryover effect of swearing. This alone is not strong evidence. However, given the knowledge that state BAS was increased in a manner consistent with the state disinhibition theory, as well as the knowledge that increased BAS should co-occur with decreased BIS, the possibility should be considered that state BIS was reduced, but that this change was not effectively captured in this study.

In addition, we hypothesised (Hypotheses 7 and 9) that ratings of positive emotion, humour, distraction, and novelty would be higher in the swearing condition compared with the neutral word condition, in line with previous research (Stephens et al., 2018, 2023). These hypotheses were confirmed for positive emotion, humour, and distraction. While not directly demonstrating state disinhibition, effects for positive emotion and humour are consistent with activation of the hot cognitions pathway of Hirsh et al.’s (2011) state disinhibition framework, while swearing effects for distraction and novelty are consistent with activation of the attention pathway of the state disinhibition framework.

While the confirmed hypotheses discussed above support our theory that state disinhibition explains how swearing boosts physical performance, not all our results were consistent with these expectations. We hypothesised (Hypothesis 3) that ERN amplitude would be reduced in the swearing condition. This hypothesis was not supported by confirmatory analyses as there was no difference in mean ERN amplitude across the swearing and neutral word conditions. Based on these results, it is not surprising that the hypothesised (Hypothesis 13) mediation of the effect swearing on strength through ERN was not significant. Our results therefore do not support an explanatory role for ERN on the link between swearing and strength performance. The absence of a clear difference in ERN between conditions can be interpreted in a number of ways. As covered more extensively in the limitations section, the absence of an effect here could be due to methodological limitations. Relatedly, it is possible that the ERN is not an effective measure for state disinhibition, either because the ERN is not actually linked to state disinhibition or because the effects of state disinhibition are too small to detect reliably in smaller studies. Indeed, studies looking at the effect of induced emotional states on the ERN have yielded inconsistent results, indicating that the effect is likely hard to reliably measure (Nuñez-Estupiñan et al., 2022).

Several other pre-registered hypotheses were not supported. We hypothesised (Hypothesis 8) decreased negative emotion following swearing, assuming that reduced BIS activity due to swearing would reduce negative emotions such as anxiety. However, a significant effect was found in the opposite direction, with participants reporting more negative emotion following swearing compared with neutral word repetition. Nevertheless, this effect is consistent with increased state disinhibition following swearing if the negative emotion experienced was anger rather than anxiety. However, as we cannot be certain this is the case, we do not make any theoretical claim based on this effect of swearing on self-rated negative emotion.

We also hypothesised that in the swearing condition we would see (Hypothesis 2) an increase in committed errors on the flanker task as well as (Hypothesis 6) increased flow, (Hypothesis 10) increased state confidence, (Hypothesis 11) decreased cognitive anxiety, and (Hypothesis 12) increased somatic anxiety. However, all of these analyses returned null main effects for swearing. The lack of difference in flanker errors could signify an absence of state disinhibition in the swearing condition, as previous studies have shown increased flanker errors in situations where state disinhibition would be likely, such as following acute alcohol consumption (Ridderinkhof et al., 2002). The absence of an effect of swearing on flow contradicts previous findings but may reflect a lower level of statistical power afforded by the sample size of the present study (*N* = 50) compared with previous studies (e.g., *N* = 118 for Stephens et al., 2023). Insufficient statistical power may also underlie the absence of effects for flanker errors, state confidence, cognitive anxiety, and somatic anxiety. The sample size was limited in this study compared with previous comparable experiments (e.g., Stephens et al., 2023) due to the resources necessary to collect EEG data.

Above, we have discussed the outcomes of the current study, both those consistent and inconsistent with our hypotheses, and what each hypothesis could tell us about the role of state disinhibition in explaining how swearing may promote physical task performance. In general, we interpret the results as providing limited support for the theory that swearing brings about state disinhibition. This limited support is based on confirmatory analyses supporting our hypotheses for the variables BAS drive, humour rating, and novelty rating that are indicatory of an inhibited state following swearing. However, some results were inconsistent with the state disinhibition theory, although these were characterised by an absence of effects, rather than effects contradicting the hypotheses. These could be explained by limitations of the study and power issues. For this reason, we weigh the significant outcomes of the study somewhat more heavily.

### Limitations

Returning to the ERN variable, one methodological limitation in the procedure for measuring it is that multiple trials of the flanker task are required to generate sufficient errors. We have a concern that the length of time on task may have outlasted any effects of swearing (the swear word was repeated prior to completing the flanker task), limiting the opportunity for swearing to impact the ERN. We could have employed more repetitions of the word articulations during the flanker task trials although then we may have run into habituation effects whereby the effect diminished with increasing numbers of articulations. Given this level of uncertainty, we suggest that further attempts to assess ERN in the context of state disinhibition due to swearing should employ a procedure that can yield a reliable ERN signal in a shorter timeframe.

As already alluded to, the methodology may further have been limited by condition order effects. We applied a within-subjects design in which the swearing versus neutral word condition order was blocked creating two halves of the study. An alternative approach would have been to apply a between-subjects design, although there is a strong precedent for applying within-subjects designs in studies on swearing and strength (Spierer et al., 2018; Stephens et al., 2023), and on swearing and pain (Robertson et al., 2017; Stephens et al., 2009; Stephens & Robertson, 2020; Stephens & Umland, 2012). We employed blocking to minimise risk of carry over effects, but perhaps we were too cautious in this regard. Unfortunately, order effects may have overshadowed potential effects the vocalisations might have had. We recommend further research on swearing articulation can continue to employ within-subjects designs but should seek to limit the overall timespan of the study procedure.

As already noted, the burden of testing participants within an EEG procedure meant that the sample size of this study was lower than previous studies on swearing and strength, possibly limiting study power for some of the variables predicted to show differences across swearing and neutral word conditions (Stephens et al., 2023).

It should further be born in mind that ERN amplitude has a limited evidential base linking it to state disinhibition. Evidence for this measure comes from studies showing reduced ERN in situations where state disinhibition is expected (Curtin & Fairchild, 2003; Easdon et al., 2005; Ridderinkhof et al., 2002), or from studies on psychopathology and trait disinhibition (Pasion et al., 2016). Without a direct precedent for this approach, it is possible that the ERN is not an effective measure for state disinhibition. Here we should also consider that mood induction studies indicate that measurable affective state effects on the ERN are not always straightforward and can produce null or even contradictory results (e.g., Nigbur & Ullsperger, 2020; Nuñez-Estupiñan et al., 2022). Given this, it remains a possibility that ERN amplitude is not meaningfully impacted by changes in state disinhibition, or that the effect is so subtle that it is drowned out by confounding factors.

## Conclusion

This pre-registered study has confirmed that repeating a swear word leads to increased performance on a grip strength task relative to a neutral word while also confirming effects of swearing on positive emotion, humour, and distraction. The study has further made the novel contribution of gathering data confirming that swearing raises BAS Drive, supporting application of Hirsh et al.’s (2011) state disinhibition framework to swearing, linked to the BIS/BAS theory (Carver & White, 1994). The study is the first to measure state disinhibition using the ERN signal of the EEG in the context of swearing, although no effect of swearing was found for ERN. We interpret a lack of ERN effect as most likely linked to methodological issues, specifically the lengthy procedure required to assess the ERN which may have outlasted the timespan for which effects of swearing remain active. On the basis of these confirmed findings, we conclude that state disinhibition may show limited utility in explaining how swearing impacts physical performance. We recommend further, higher powered research for confirming links between swearing, state disinhibition, and state BIS/BAS activation. It would also be useful to gain understanding of how long acute effects of repeating a swear word for a given period of time last.

## Supplemental Material

sj-docx-1-qjp-10.1177_17470218241308560 – Supplemental material for The effect of swearing on error-related negativity as an indicator for state disinhibition Supplemental material, sj-docx-1-qjp-10.1177_17470218241308560 for The effect of swearing on error-related negativity as an indicator for state disinhibition by Venja Beck, Joseph L Brooks and Richard Stephens in Quarterly Journal of Experimental Psychology
