# Supplementary material for: The effect of swearing on error-related negativity as an indicator for state disinhibition
Source: Q J Exp Psychol (Hove). 2025 Jan 13;78(11):2390–402. doi: 10.1177/17470218241308560 (PMC12531393; doi:10.1177/17470218241308560)
Supplement: sj-docx-1-qjp-10.1177_17470218241308560 – Supplemental material for The effect of swearing on error-related negativity as an indicator for state disinhibition [file sj-docx-1-qjp-10.1177_17470218241308560.docx]

The Effect of Swearing on Error-Related Negativity as an Indicator for State Disinhibition.

Venja Beck (2), Joseph Brooks (1), Richard Stephens (1)

# Supplementary materials

## Exploratory Analyses

The effect of swearing on ERN was not found to be significant. However, in exploratory analyses in which the order of conditions was taken into account we do see a significant interaction effect between condition order and condition on the ERN, *F*(1, 43) = 5.744, *p*=0.021, η_p_^2^ = 0.118. In both cases we see a higher mean amplitude for the condition that came first versus the condition that came second. Descriptives are plotted in Figure S1. A split-half analysis was conducted to comparing conditions for the trials most closely following word repetition, but this provided no novel insights.

There was a significant interaction between state BIS scores and condition order, *F*(1,48) = 5.601, *p* = 0.022, *ηp^2^* = 0.105. The descriptive plot in S2 and contrasts indicate an overall order effect (*p* = 0.022), as well as a difference between conditions when swearing was the second condition (*p* = 0.005; lower BIS score in the swearing condition) but no difference between the conditions when swearing was the first condition.

To further consider the link between BAS Drive, Swearing, and strength, an exploratory analysis of the effect of swearing on strength mediated through BAS Drive was conducted, shown in figure S3. This analysis was not preregistered. While the effects of swearing on strength and swearing on BAS drive were both significant, mediation analysis showed no support for the mediated pathway.

**
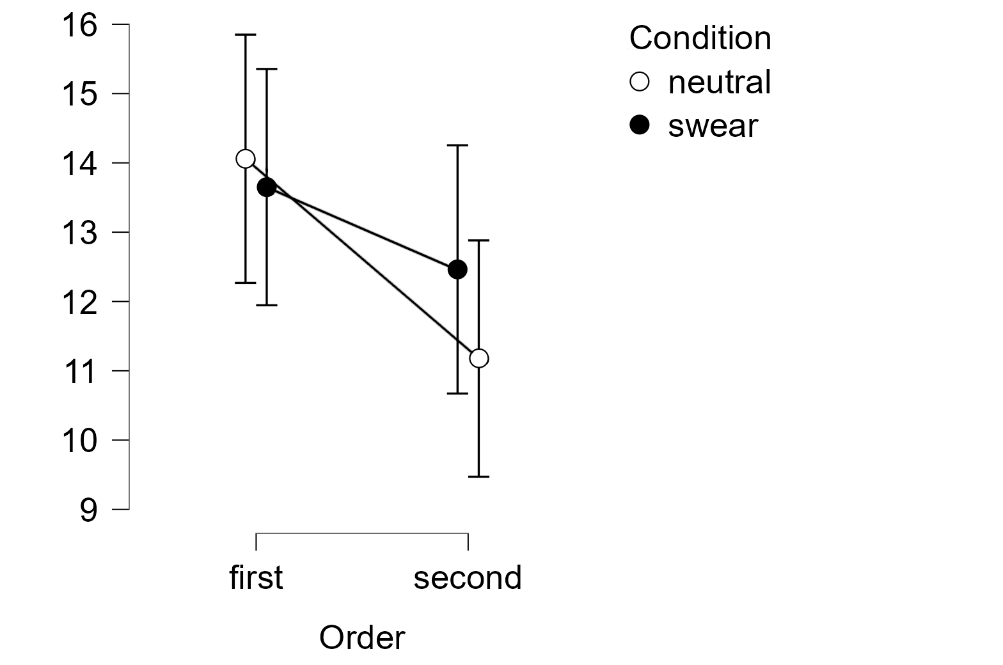
**

**Figure S1.** mean ERN amplitude in µV by first condition (horizontal axis) and condition (white dots for swearing, black dots for neutral).


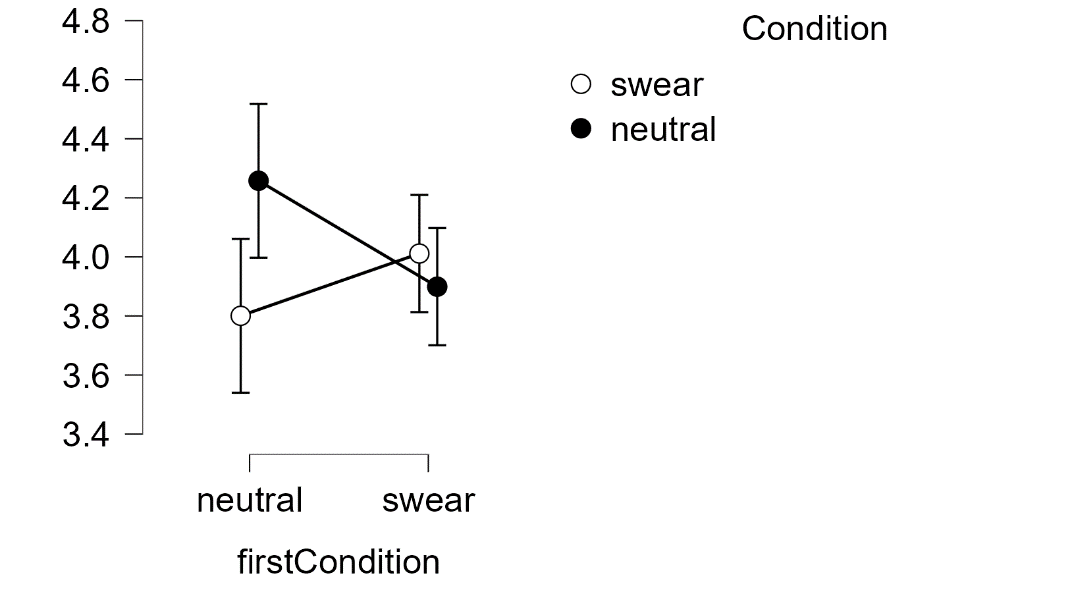


**Figure S2**. Mean BIS by first condition (horizontal axis) and condition (white dots for swearing, black dots for neutral).


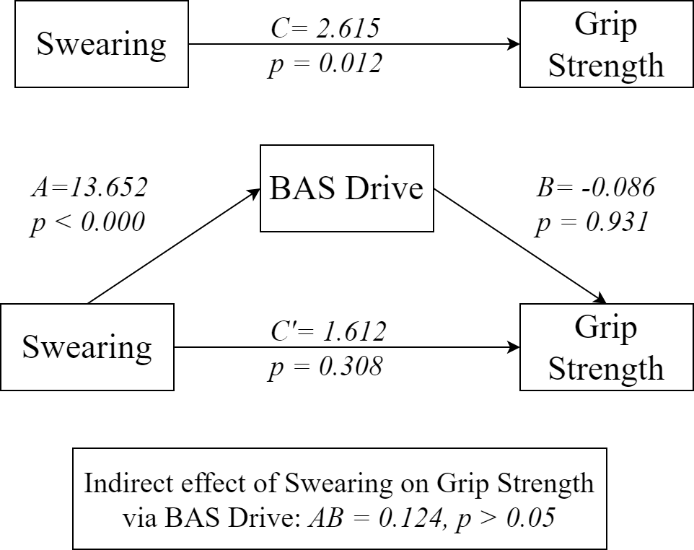


**Figure S3.** BAS drive (exploratory) (right)
